# Data for discriminating dead/live bacteria in homogenous cell suspensions and the effect of insoluble substrates on turbidimetric measurements

**DOI:** 10.1016/j.dib.2017.04.003

**Published:** 2017-04-08

**Authors:** Kwabena O. Duedu, Christopher E. French

**Affiliations:** a Institute of Quantitative Biology, Biochemistry and Biotechnology, School of Biological Sciences, University of Edinburgh, Edinburgh EH9 3FF, UK; b Department of Biomedical Sciences, School of Basic & Biomedical Sciences, University of Health & Allied Sciences, Ho, Ghana

**Keywords:** Bacteria growth, Fluorimetry, Live/dead estimation, Propidium iodide, SYBR Green I nucleic acid gel stain

## Abstract

Estimation of bacterial growth by rapid traditional methods such as spectrophometric measurements at 600 nm (OD600) is not applicable for cultures containing insoluble particles in the growth media. Colony counts are the only suitable alternative but these are laborious and not high-throughput. The data presented in this article is related to the research article entitled “Two-colour fluorescence fluorimetric analysis for direct quantification of bacteria and its application in monitoring bacterial growth in cellulose degradation systems” (Duedu and French, 2017) [1]. This data article presents original primary data describing the discrimination of dead/live bacteria in homogenous cell suspensions and how the presence of insoluble substrates affect the turbidity of the suspensions.

**Specifications Table**

**Table:** 

Subject area	*Applied Microbiology*
More specific subject area	*Bacterial growth and quantification*
Type of data	*Table and figure*
How data was acquired	*Absorbance at 600 nm was acquired using the absorbance module on the Turner BioSystems’ Modulus™ Single Tube multimode reader whereas fluorescence was measured after staining with SYBR-I and/or propidium iodide (PI) using various fluorescent modules on the same device; Ultra-sonication of cells was done using the MSE Soniprep 150 Plus Ultrasonic Disintegrator.*
Data format	*Raw data collection and analysis*
Experimental factors	*Cell lysis and nucleic acid staining*
Experimental features	*Overnight cultures of bacteria were serially diluted and lysed using ultra-sonication, then nucleic acid stained with SYBR-I and PI to differentiate live and dead cells. Degree of cell damage was assessed by protein estimation with BSA standards. 3 biological replicates were used.*
Data source location	*Edinburgh, United Kingdom*
Data accessibility	*Data is presented in this article*

**Value of the data**

• The data describes background fluorescence in red and green fluorescence channels for SYBR-I and PI stained cells/DNA respectively. This is important as it gives an idea of the contribution of the background fluorescence to actual fluorescence and thus helps to determine corrective measures.
• Ultra-sonication has been explored as a rapid way of inducing cell damage in bacteria which can be used to evaluate many applications such as discrimination of live and dead cells which has been presented here.
• Presence of insoluble substrates affecting turbidimetric measurement (OD600) and allowing samples to settle for up to 15 minutes does not correct this.

## 1 Data

**Fig. 1:**
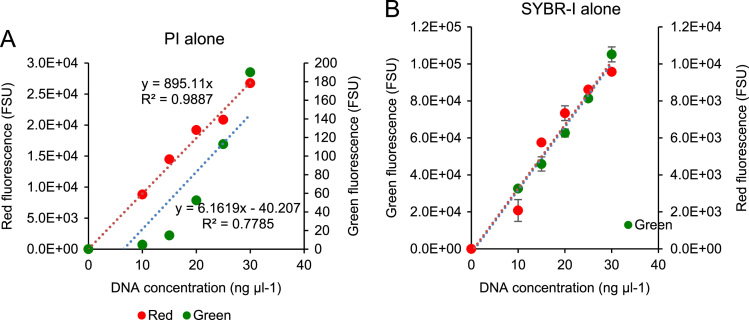
Background fluorescence of PI (left) and SYBR-I (right) in green and red channels respectively. Plots represent means of three biological replicates.

The data shown here represents a systematic process of inducing and assessing cell damage as well as the contribution of the presence of insoluble substrates to the optical density measurement of cell suspensions at 600 nm and enumeration of live cells following dual staining with SYBR Green I nucleic acid gel stain and propidium iodide. Turbidimetric measurements (optical density at 600 nm, OD600) and viable (plate/colony) counts [2], [3] are the commonly used methods for direct quantification of bacteria. Fig. 1 shows background green and red fluorescence produced by the two stains in each other׳s fluorescence excitation and emission channel. The overlapping excitation and emission profiles responsible for the background fluorescence was reported in the research article related to this data [1].

**Fig. 2:**
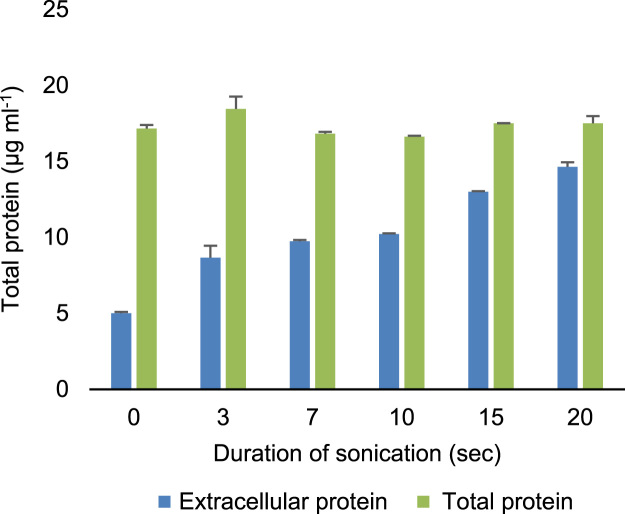
Extracellular protein increases with increasing ultra-sonication demonstrating that ultra-sonication damages cells. The number of dead cells as a result of the ultra-sonication as determined by fluorescence staining correlate with the release of extracellular protein estimated using the Pierce Coomassie Plus (Bradford) Assay (ThermoScientific, Rockford, lL, USA) (Fig. 3).

Fig. 2 demonstrates cell damage induced by ultra-sonication and evaluated by estimation of the total protein content of the cell suspensions. The data demonstrates that ultra-sonication of cells induces varying degrees of cell damage directly proportional to the duration of ultra-sonication representing increasing cell lysis and release of protein. However, when the same samples were totally lysed by incubation at 65 °C (1 h), the total protein was not significantly different from each other.

**Fig. 4:**
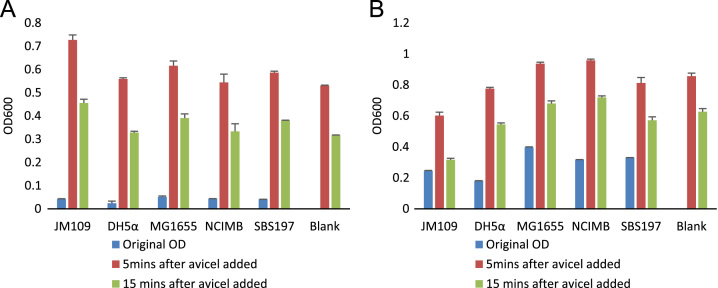
Changes in turbidity of cell suspensions with the addition of insoluble cellulosic substrate. *Escherichia coli* strains JM109, DH5α and MG1655 and *Citrobacter freundii* strains NCIMB11490 and SBS197 were used. ‘A’ shows results from cell suspensions with lower densities than those in ‘B’. Error bars are standard errors of three biological replicates.

Addition of cellulose significantly increased the OD600 of cell suspensions and this increase (noise) was not cleared even when suspensions were allowed to stand for up to 15 min (Fig. 4). This observation was the same for all the different strains of bacteria used. Furthermore, a similar observation was made for suspensions with higher or lower numbers of cells.

**Fig. 5:**
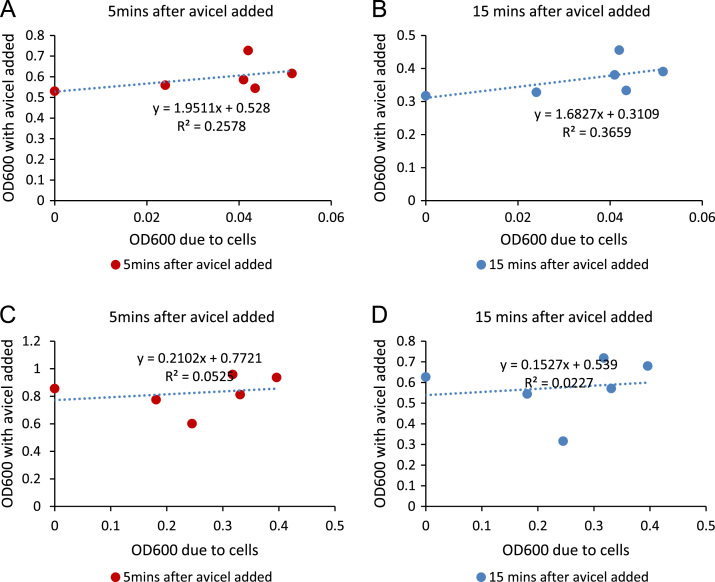
Correlation between OD600 before and after addition of cellulose and its settling.

To test whether the presence of an insoluble substrate will only add proportional increase to the OD600 or not, cellulose was added to cell suspensions. There was no correlation between the original OD600 and the OD600 when insoluble substrates are present (Fig. 5). The correlation was not improved even when the cell suspensions were left to stand for 5 min or 15 min.

## 2 Experimental design, materials and methods

Human genomic DNA (200 ng µl^-1^) was obtained from Bioline, London, UK, diluted serially in nuclease free water and used as a calibration standard. The diluted standards were stained with SYBR-I and/or PI and the fluorescence was measured using the Modulus™ Single Tube multimode reader. The blue (P/N 9200-040, λ_ex_ = 460 nm, λ_em_ = 515-570 nm) or green (P/N 9200-042, λ_ex_ = 525 nm, λ_em_ = 580–640) Modulus™ fluorescence kits (Turner BioSystems, Sunnyvale, CA, USA) were used for the measurement of green and red fluorescence respectively. Cell density was determined by measuring absorbance of cell suspensions at 600 nm using the absorbance module (Model E6076, GLOMAX MultiJR, Promega, Southampton, UK) on the Modulus reader.

To induce cell damage and evaluate how useful dual staining with SYBR-I and PI is for quantification of live bacterial cells demonstrated in flow cytometry [4], [5], [6], [7], [8], [9], [10] and demonstrated for fluorimetry in ref [1], a cell suspension was prepared and aliquoted into different tubes numbered 1 to 6. The tubes (1 to 6) were ultra-sonicated for one pulse at 10 µm (amplitude) for 0, 3, 7, 10, 15 and 20 s each respectively on ice using the MSE Soniprep 150 Ultrasonic Disintegrator. The degree of damage was accessed by the quantity of extracellular protein using the Pierce Coomassie plus (Bradford) assay kit (ThermoScientific, Rockford, lL, USA) following the manufacturer׳s instructions. Dual staining of the ultra-sonicated cells with SYBR-I and PI was performed to determine whether damage to the cells correlated with dead cells as estimated from fluorescence measurements.

To determine the effect of the presence of an insoluble substrate in the cell suspension, the suspensions were spiked with equal amounts of microcrystalline cellulose (avicel). The OD600 was determined. Suspensions were also left to stand for 5 and 15 minutes to check whether OD600 values or the live or dead cell quantification will be improved. Dual staining with SYBR-I and PI for fluorimetry has been described in the research article related to this data [1].

## Figures and Tables

**Fig. 3:**
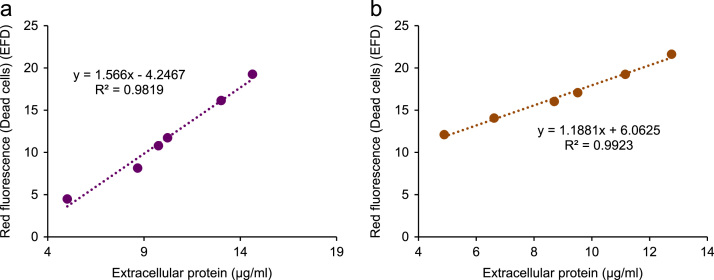
Strong correlation between the dead cells estimated by red fluorescence following dual staining of both *E. coli* (A) and *C. freundii* (B).

## References

[1] Duedu K.O., French C.E. (2017). Two-colour fluorescence fluorimetric analysis for direct quantification of bacteria and its application in monitoring bacterial growth in cellulose degradation systems. J. Microbiol. Methods.

[2] Breed R.S., Dotterrer W.D. (1916). The number of colonies allowable on satisfactory agar plates. J. Bacteriol..

[3] Koch A.L. (1970). Turbidity measurements of bacterial cultures in some available commercial instruments. Anal. Biochem..

[4] Kaprelyants A.S., Kell D.B. (1992). Rapid assessment of bacterial viability and vitality by rhodamine 123 and flow cytometry. J. Appl. Bacteriol..

[5] Porter J., Deere D., Pickup R., Edwards C. (1996). Fluorescent probes and flow cytometry: new insights into environmental bacteriology. Cytometry.

[6] Barbesti S., Citterio S., Labra M., Baroni M.D., Neri M.G., Sgorbati S. (2000). Two and three-color fluorescence flow cytometric analysis of immunoidentified viable bacteria. Cytometry.

[7] Diaper J.P., Tither K., Edwards C. (1992). Rapid assessment of bacterial viability by flow cytometry. Appl. Microbiol. Biotechnol..

[8] Caron G.N.-V., Badley R.A. (1995). Viability assessment of bacteria in mixed populations using flow cytometry. J. Microsc..

[9] Foladori P., Bruni L., Tamburini S., Ziglio G. (2010). Direct quantification of bacterial biomass in influent, effluent and activated sludge of wastewater treatment plants by using flow cytometry. Water Res..

[10] Tamburini S., Foladori P., Ferrentino G., Spilimbergo S., Jousson O. (2014). Accurate flow cytometric monitoring of Escherichia coli subpopulations on solid food treated with high pressure carbon dioxide. J. Appl. Microbiol..

